# Supplementary material for: Migration in the social stage of Dictyostelium discoideum amoebae impacts competition
Source: PeerJ. 2015 Oct 22;3:e1352. doi: 10.7717/peerj.1352 (PMC4627915; doi:10.7717/peerj.1352)
Supplement: Supplemental Information 1 [file peerj-03-1352-s001.docx]

**Pre-experiment clone RFP-fluorescent stability**

In order to be used for the experiment *D. discoideum* clones had to meet three requirements: 1) they had to have a high transformation efficiency rate; 2) they needed to remain stable for 1 week without selection; and 3) they could not cheat or segregate from their wild-type ancestor.

SF1. The proportion of each clone that was labeled before and after development.

SF2. The proportion of the fluorescent clone and its ancestor before and after development

**Description of raw data spreadsheet**

The spreadsheet for the raw data is divided into four tabs- two that show the data on spore production and cheating and two that show the morphometric data for the fruiting body architecture. Below I describe the columns that are raw data plus some columns with minimum manipulation that are needed to understand how we obtained our results.

Tab 1 is named **sporeMig-clonal**. This shows the spore production and distance traveled data for the clonal groups.

- The first four columns are descriptive, giving the clone, whether it was transformed or not (RFP or Anc), and replicate number (1-5)
- **Cell number** is the number of cells that were pipetted onto the agar plates.
- **Spore number** is the number of spores that we collected and counted at the end of the experiment.
- **Sporulation efficiency** is Spore number divided by Cell number
- **Zones 0-6** are the number of fruiting bodies counted in each zone
- **Total Fruiting bodies** is the sum of the zone columns
- **Average Distance** is the calculated average distance traveled by the slugs on the plate.

Tab 2 is named **sporeMigChimeras**. This shows spore production and distance traveled for the chimeric groups.

- The first six columns are the same as for the clonal tab but it has information for both clones used in the chimera.
- **Cell.mix.no** is the number of cells that were pipetted onto the agar plates.
- **Spore.mix.no** is the number of spores that we collected and counted at the end of the experiment.
- **total sporulation efficiency** is Spore.mix.no divided by Cell.mix.no
- **clone 1 sporulation efficiency** is the proportion of clone 1’s spore number to cell number in the mix
- **clone 2 sporulation efficiency** is the proportion of clone 2’s spore number to cell number in the mix
- **columns L-O**  are a check of the proportion of red cells in the transformed clone (clone 1) only. It will be used to determine the overall proportion of clone 1 to clone 2 after adjusting for cells of clone 1 that did not fluoresce
- **columns P/Q** are the cells that we counted as red or not red.
- **Column S (c.mix.prop.red)** is the unadjusted proportion of red (clone 1) cells in the mix
- **Column U (c.mix.prop.adj)** is the proportion of red (clone 1) cells in the mix after adjusting for cells of clone 1 that did not fluoresce
- **Columns V-AD** are the same as the last three columns from the sporeMigClonal tab. They show the average distance traveled by slug per plate.
- **Columns AE-AN** are the same as columns L-U, except they are counts for spores instead of cells.

Tabs 3 and 4 have the same column headings. Tab 3 the morphometric measurements for the clonal fruiting bodies. Tab 4 has the measurements for the chimeric fruiting bodies.

- **Column B** is the length of the stalk in pixels
- **Columns C, D, and E** are the widths of the stalk at the top, middle, and bottom in pixels
- **Columns J and K** are the conversion factor between pixels and um.
- **Columns L-N** are the stalk measurements and sorus width converted into um
- **Columns O and P** are the volumes of the fruiting bodies in um
